# Artificial Sweeteners in Food Products: Concentration Analysis, Label Practices, and Cumulative Intake Assessment in Croatia

**DOI:** 10.3390/nu17071110

**Published:** 2025-03-22

**Authors:** Zlatka Knezovic, Branka Jurcevic Zidar, Ajka Pribisalic, Sanja Luetic, Katarina Jurcic, Nina Knezovic, Davorka Sutlovic

**Affiliations:** 1 Teaching Institute for Public Health, Split-Dalmatia County, 21000 Split, Croatia; zlatka.knezovic@nzjz-split.hr (Z.K.); branka.jurcevic.zidar@nzjz-split.hr (B.J.Z.); sanja.luetic@nzjz-split.hr (S.L.); katarina.jurcic@nzjz-split.hr (K.J.); nina.knezovic@nzjz-split.hr (N.K.); 2 Department of Health Studies, University of Split, 21000 Split, Croatia; dsutlovic@ozs.unist.hr; 3 Department of Public Health, School of Medicine, University of Split, 21000 Split, Croatia; 4 Department of Applied Pharmacy, School of Medicine, University of Split, 21000 Split, Croatia

**Keywords:** artificial sweeteners, foods analysis, acceptable daily intake, cumulative consumption, public health

## Abstract

Background/Objectives: Artificial sweeteners (ASs) are food additives used to impart sweetness to various food products. Common sweeteners used individually or in combination include acesulfame-K, aspartame, cyclamate, saccharin, sucralose, and neotame. While traditionally considered harmless, emerging research suggest potential health implications. This study aims to analyze commonly consumed food products in Croatia for ASs presence, quantify four ASs, and estimate daily intake of ASs. Additionally, product labeling was assessed for compliance with Regulation 1169/2011 on food information to consumers. Methods: This study assessed the presence of acesulfame-K, aspartame, cyclamate, and saccharin dihydrate in 121 frequently consumed food products from the Croatian market using a high-performance liquid chromatography method. Based on obtained concentrations, data from a parallel consumption study, and existing literature on acceptable daily intake (ADI), we assessed exposure to ASs. Results: ASs were found in a substantial proportion of analyzed products, with multiple sweeteners often present in a single product. Specifically, ASs were detected in 74% of carbonated drinks, 54% of fruit juices, 86% of energy drinks, 70% of high-protein milk products, and 66% of chewing gums. Hypothetical consumption scenarios demonstrated that children, due to their low body mass, are at the highest risk of exceeding ADI values. Conclusions: The widespread presence of ASs in food products raises concerns about excessive intake, particularly among children who frequently consume soft drinks, instant beverages, and protein drinks. These findings highlight the need for further research into cumulative ASs exposure and its potential health effects, as well as the importance of public health strategies to regulate ASs consumption.

## 1. Introduction

The increasing prevalence of overweight and obesity in contemporary life has become one of the most pressing public health concerns worldwide. Current data reveal that 29% of children in Europe are overweight or obese, while up to 53% of adults have a body mass index (BMI) above 25 [1,2]. Numerous scientific evidence indicates the impact of excess body weight on the development of chronic non-communicable diseases, which are now leading causes of death globally [3]. Despite extensive public health recommendations for proper nutrition, a significant proportion of the population across all age groups continues to consume high levels of sugar, adversely affecting their overall nutritional status. The World Health Organization guidelines recommend that sugar intake should be reduced to less than 10% of the total daily calorie intake for both children and adults, as a measure to mitigate the risk of developing obesity and dental caries [4].

Given the rising concerns over excessive sugar consumption and its impact on health, artificial sweeteners (ASs) emerged as a promising alternative. With their intense sweet taste and negligible caloric content, these additives allowed for the creation of foods that maintain desirable sweetness and sensory qualities while reducing overall caloric intake. As a result, over time, the range of food products incorporating ASs, either individually or in combinations, has significantly expanded [5,6,7,8,9,10].

To ensure safety, all food additives, including ASs, undergo a thorough safety assessment evaluation by the European Food Safety Authority (EFSA) before being authorized for use [11]. Additives approved before 2009 must also be reassessed in light of the latest scientific evidence [12]. A key aspect of this safety assessment involves estimation of dietary exposure and establishing an acceptable daily intake (ADI). In the European Union, 17 sweeteners and their combinations are currently approved. Their use is regulated by Regulation (EC) No. 1333/2008 of the European Parliament and of the Council on food additives, which outlines the food categories where these sweeteners can be added and specifies their maximum permitted amounts [13].

According to the latest data from 15 November 2024, the EFSA panel on Food Additives and Flavorings published a re-evaluation of saccharin and its salts as food additives, concluding that saccharin is safe for human consumption. Consequently, the ADI for saccharin has been increased from 5 to 9 mg kg^−1^ of body weight [14]. Currently, saccharin (E 954) is an approved food additive in the EU across 34 food categories, with maximum permitted levels ranging from 50 to 3000 mg kg^−1^. Additionally, it is authorized for use at quantum satis (QS) in three food categories, including tabletop sweeteners in liquid, powder, and tablet form. Meanwhile, the EFSA is in the process of re-evaluating the safety of acesulfame-K and cyclamate [15].

**Figure 1:**
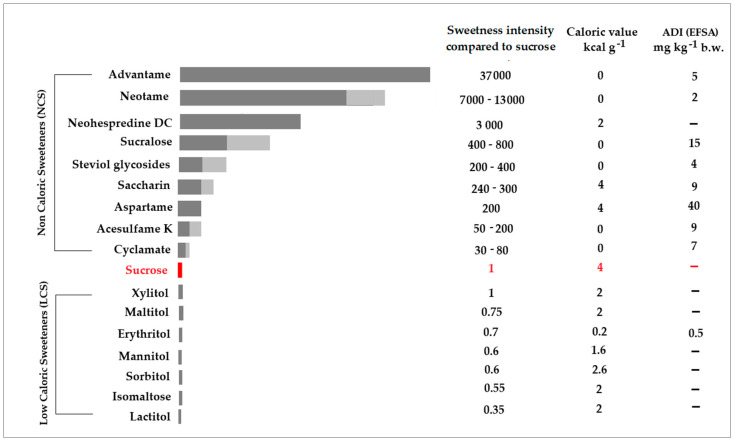
Characteristics of various artificial sweeteners, including their relative sweetness, caloric value, and acceptable daily intake (ADI). Light and dark gray represent approximate ranges of AS sweetness relative to sucrose, which is marked in red.

The evolving regulatory landscape surrounding ASs underscores the need for a clear understanding of their classification and properties. Based on their caloric content, these sweeteners are typically classified as Non-Caloric Sweeteners (NSCs) and Low-Caloric Sweeteners (LSCs). The characteristics of these sweeteners, including their relative sweetness compared to sucrose, caloric values, and ADIs, are summarized in Figure 1.

ASs can be incorporated into a broad range of food products, including flavored fermented milk products and ice cream, fruit nectars, flavored drinks, canned or bottled fruit and vegetable preparations, jam, jellies and marmalades, cocoa and chocolate products, and confectionaries. Additionally, they are commonly found in chewing gums; breakfast cereals; fine bakery wares, potato-, cereal-, flour-, or starch-based snacks; processed nuts; processed fish and fishery products; soups and broths; sauces; and salad spreads. Their use also extends to alcoholic beverages, such as liqueurs, alcohol-free beer, food supplements, dietary foods for special medicinal purposes, and dietary foods intended for weight control [13].

This widespread use of ASs complicates the assessment of dietary exposure. Typically, food consumption data are gathered through surveys conducted across different age groups [16]. However, to acquire accurate and precise data, it is assumed that the respondents are well-informed of food properties. Consumers primarily receive product information through labeling, which is regulated by Regulation 1169/2011 on the provision of food information to consumers [17]. This regulation mandates that all necessary information must be presented clearly, unambiguously, and in a visible and legible manner. Additionally, the regulation emphasizes that in food with sweeteners, the statement “with sweetener(s)” shall accompany the name of the food. Despite these requirements, most often, information about the presence of sweeteners is not stated in a conspicuous place, but on the back, a less visible part of the label. At the same time, marketing elements, such as claims about vitamins, minerals, or other beneficial ingredients, are typically displayed in the principal field of vision on the product packaging. This practice of less conspicuous labeling, combined with findings from various surveys, reveals that many consumers rarely read the back of products. Moreover, consumers are often unaware of the distinctions between the subtypes in product categories, leading to purchase decisions influenced more by product name and image than by detailed label information [18,19,20,21].

In response to numerous public health campaigns aimed at reducing sugar intake, manufacturers have increasingly substituted part of the sugar with ASs [22,23]. Therefore, the use of ASs in the food industry is increasing every day, presenting a significant challenge in accurately assessing the actual dietary exposure. Also, the health impacts of ASs are still a topic of considerable debate. Numerous studies have reported contradictory results, ranging from potential beneficial health effects to various adverse effects on human health [24,25,26,27,28,29,30]. These concerns underscore the importance of accurately assessing dietary exposure to ASs to better understand their potential health effects.

Therefore, the aim of this study was to analyze the most commonly consumed soft drinks and other food products from Croatian markets for the presence of ASs and to measure the concentrations of four ASs in order to calculate the estimated daily intake of ASs. Additionally, one of the objectives was to assess product labeling of such products and evaluate whether the declaration of ASs complies with the requirements of Regulation 1169/2011 on the provision of food information to consumers.

## 2. Materials and Methods

### 2.1. Respondents

An anonymous survey was conducted during March and April 2024 among two groups of respondents: parents of preschool and school-age children and adults, including university and secondary school students.

Respondents were recruited through a combination of online and in-person approaches. Parents of preschool and school-age children were identified through enrollment records from two urban kindergartens and one elementary school. These institutions were selected based on agreements with school administrators and kindergarten directors, who facilitated survey distribution. Invitations containing a link to the online survey were shared via parent–teacher associations and school mailing lists. University and secondary school students were recruited from a population including medical, pharmacy, health sciences, and other university students (natural sciences, technical sciences, and social sciences), as well as senior students from grammar and vocational schools. Participants were invited through university communication channels and student networks.

Participation was voluntary, and all respondents provided informed consent before completing the survey. The first survey was completed by a total of 324 parents of preschool and school-age children [31]. The second survey was completed by a total of 325 university and secondary school students, and results are provided in the previous paper [32].

This study was conducted in accordance with the Declaration of Helsinki, and the protocol was approved by the Ethics Committee of University Department of Health Studies, University of Split (Class: 029-03/24-18/01) on 1 March 2024.

### 2.2. Samples

Based on the evaluation of the questionnaires from our studies, 121 samples—including a variety of beverages, chewing gums, and other food products regularly purchased and consumed by the respondents—were sampled and analyzed. The food product samples were collected in May and June 2024 and analyzed to determine the composition and concentrations of four ASs: acesulfame-K, aspartame, saccharine, cyclamate. The samples were categorized into seven groups: carbonated and non-carbonated soft drinks (n = 47), fruit juices/nectars (n = 26), syrups for dilution (n = 3), instant drinks (n = 6), energy drinks (n = 7), high-protein milk products (n = 20), and chewing gums (n = 12). Information on the types and quantities of products consumed was obtained from the survey results.

### 2.3. Methods

The determination of acesulfame potassium, sodium saccharin dihydrate, and aspartame was conducted in accordance with HRN EN 12856:2000 [33], while cyclamate was analyzed following the HRN EN 12857:1999 procedure [34].

The analyses were performed using an Agilent 1200 HPLC system (Agilent Technologies Singapore (International) Pte. Ltd., Singapore) equipped with a diode array detector (190–400 nm) and OpenLab CDS Chemstation Software (Agilent, Singapore, rev. B.04.03), as well as an Agilent 1260 HPLC system (Agilent Technologies Singapore (International) Pte. Ltd., Singapore) featuring a diode array detector (190–900 nm) and OpenLab CDS Chemstation Software (Agilent, Singapore, rev.C.01.05).

The limit of quantitation (LOQ) for all four analytes was 2.5 mg L^−1^. The mean recovery was ≥95.0%, with an excellent linearity (R^2^ = 0.999) observed for all analytes. Each sample was analyzed in duplicate, yielding a relative standard deviation of less than 5%. The results are reported as mean values.

A detailed description of sample preparation and analytical procedures for determination of acesulfame potassium, sodium saccharin dihydrate, aspartame, and cyclamate is described in our previous study [31].

### 2.4. Statistical Analysis

The concentrations of ASs are presented as arithmetic averages and ranges. The calculations were performed using a Microsoft Excel (Microsoft Office LTSC Professional Plus 2021).

## 3. Results

This study included a total of 649 participants, comprising preschool and school-aged children (N = 324) and university and secondary school students (N = 325). The overall sample consisted of 40.2% females and 59.8% males, with a median age of 18.0 years (IQR: 16.0) and a median BMI of 18.93 (IQR: 6.98). Among preschool and school-aged children, the gender distribution was 44.8% female and 55.2% male, with a median BMI of 15.38, while university and secondary school students were predominantly female (74.8%), with a median BMI of 22.15. The educational background of the university and secondary school students in the study varied, with 47.4% from Biomedical Sciences, followed by 8.3% from Natural Sciences, and 18.8% attending grammar secondary schools.

Parental demographic data showed that 88.9% of respondents were mothers, with an average age of 37.1 years and a median BMI of 22.94. Educational attainment was relatively high, with 51.9% of parents holding a university degree and 7.7% a master’s or doctoral degree.

Regarding lifestyle factors, 34.4% of all participants engaged in physical activity three or more times per week, 21.0% exercised twice a week, 8.5% once a week, and 36.2% reported no physical activity.

### 3.1. Analysis of Artificial Sweetener Concentrations Across Commonly Consumed Food Products

The results of the analyses for the four types of ASs in the sampled food products, including statistical variables such as average and concentration ranges, are presented in Table 1.

A total of 121 food products were analyzed, selected based on previous literature and market research indicating a potential presence of ASs. These products correspond to items frequently reported by respondents in questionnaires as being regularly purchased and consumed [32]. The majority of the samples (n = 89) were from a category of various types of beverages, which have been identified in multiple studies as major contributors to body weight disorders, often containing ASs as partial sugar substitutes. The analyzed beverages included carbonated and non-carbonated drinks, fruit-based drinks, and products used to prepare non-alcoholic beverages, such as syrups and instant drink mixes.

The analysis revealed that 60 of the beverages (67.4%) contained one or more ASs. The most common sweetener was acesulfame-K, which was found in 37 (61.7%), followed by cyclamate in 22 (36.7%), aspartame in 19 (31.7%), and saccharin in 18 (30.0%) of analyzed drinks. Additionally, sucralose—a widely used sweetener—was declared on labels of 22 (36.7%) of the beverages analyzed in this study.

Most beverages contained multiple types of sweeteners, a common practice to enchase the overall and achieve an optimal taste profile. This approach effectively masks undesirable properties, such as bitterness or sourness, associated with certain sweeteners [35]. Additionally, the use of multiple sweeteners allows manufacturers to achieve the desired sweetness without exceeding the individual maximum permitted levels specified by Regulation 1333/2008 on food additives. In some samples, the concentrations of individual ASs were just below the prescribed limit, although other sweeteners were also present in the same product.

**Figure 2:**
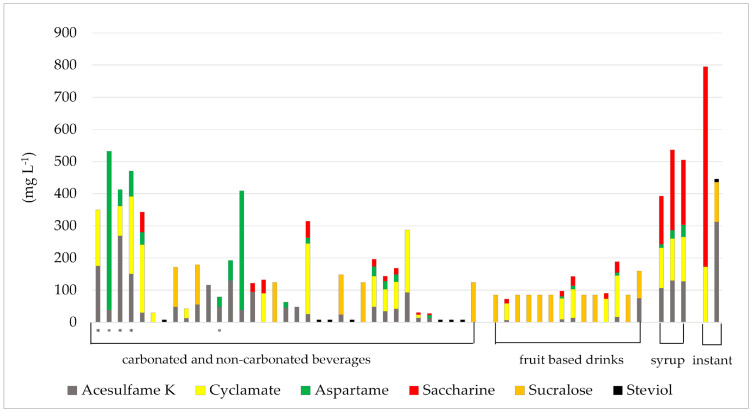
Distribution of different artificial sweeteners in carbonated and non-carbonated beverages, fruit-based drinks, syrups, and instant products analyzed in this study, including only samples in which artificial sweeteners were detected. * Indicates samples labeled as zero sugar.

The distribution of ASs in carbonated and non-carbonated drinks, fruit-based drinks, as well as syrups and instant products is shown in Figure 2. It is important to note that only the samples containing ASs are included in this analysis.

Although sucralose is commonly used as a sweetener, its content could not be measured in this study. To provide a more accurate representation of the presence and proportion of various ASs, we utilized average sucralose values reported in previous studies that analyzed similar food products. The average sucralose concentration used for the group of soft drinks was 124 mg L^−1^, while for fruit-based drinks, it was 80 mg L^−1^ [36,37,38,39,40,41] (Figure 2).

**Figure 3:**
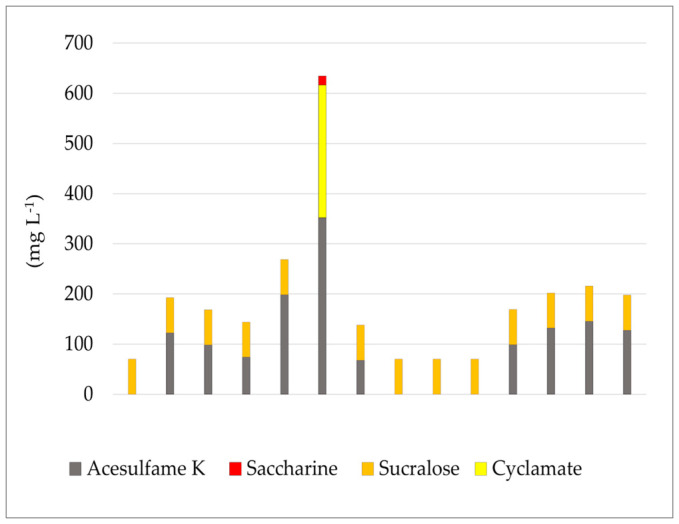
Distribution of different artificial sweeteners in high-protein milk products analyzed in this study, including only samples in which artificial sweeteners were found.

A total of 20 high-protein milk products (drinks, puddings, skyr products) were analyzed, of which 14 contained one or more sweeteners (Figure 3). The most common combination was acesulfame-K and sucralose, found in 9 out of 14 sweetened samples. One high-protein pudding sample contained three ASs, two of which, acesulfame-K and cyclamate, were present at concentrations exceeding the maximum permitted levels set by Regulation 1333/2008. Since sucralose was not directly analyzed in this study, an average value of sucralose corresponding to 70 mg L^−1^ was used for the group of milk products, based on data from several previous studies [36,39] (Figure 3).

**Figure 4:**
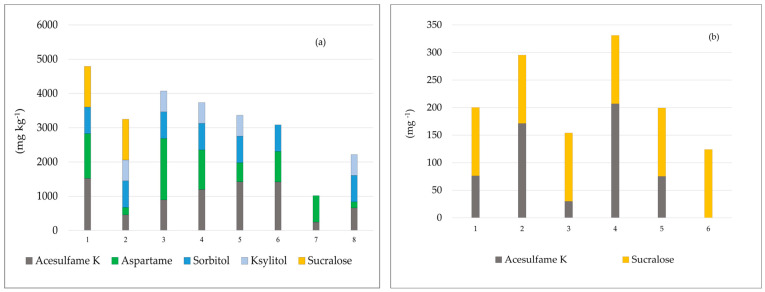
Distribution of different artificial sweeteners in samples analyzed in this study, including only samples in which artificial sweeteners were found: (**a**) chewing gums; (**b**) energy drinks.

The majority of the analyzed chewing gum samples (n = 8; 66.7%) contained combinations of multiple NCS (acesulfame-K, aspartame, and sucralose) as well as several LCS (sorbitol and xylitol). Since not all ASs could be analyzed in this study, average values for sucralose (1185 mg kg^−1^), sorbitol (608 mg kg^−1^), and xylitol (770 mg kg^−1^) were obtained from the results of similar studies to provide a clearer representation of their prevalence [40,41,42] (Figure 4a).

In six samples of analyzed energy drinks, K and sucralose were added; acesulfame-K was found in a concentration range of 30–207 mg L^−1^, while for the sucralose concentrations, we used data from similar studies (124 mg L^−1^) [36,37,38] (Figure 4b).

### 3.2. Calculation of Artificial Sweeteners Intake Based on Varying Consumption Levels

In a parallel study, parents of preschool and school-aged children [31], as well as adult university and secondary school students [32] were surveyed to assess their consumption habits of various types of beverages. Based on the reported beverage types and quantities consumed, the potential daily intake of ASs was calculated. These calculations were performed using the average and maximum concentrations of ASs obtained from the analysis of the sampled products (Table 2).

Based on the data presented in Table 2 and the consumption patterns obtained from the questionnaires [31], daily intake estimates of ASs were calculated. Three different scenarios were developed to account for variations in the type and quantity of food consumed. The results are presented in Table 3, Table 4 and Table 5.

For beverages and high-protein milk products, the ASs content was adjusted to the corresponding volume in each scenario. For chewing gum, the ASs content was calculated per piece, assuming an average mass of 1.4 g (based on the package information, e.g., 60 pieces weighing 84 g). For each AS analyzed, intake calculations were performed using both the average concentration ($\bar{x}$) and the maximum concentration (Max).

***Scenario 1*** estimates daily intake of ASs based on the daily consumption of 0.2 L of various beverages and 0.15 L of high-protein products, with no intake of chewing gum or energy drinks. The results are presented in Table 3.

***Scenario 2*** estimates daily intake of ASs based on daily consumption of 0.5 L of various beverages, 0.3 L of high-protein products, and 1 piece of chewing gum, with no intake of energy drinks. The results are presented in Table 4.

***Scenario 3*** estimates daily intake of ASs based on daily consumption of 0.5 L of various beverages, 0.5 L of high-protein products, two pieces of chewing gum, and 0.2 L of energy drinks. The results are presented in Table 5.

The ADI represents the maximum amount of a substance that can be consumed daily over a lifetime without posing a health risk, relative to a person’s body mass. Since this study included different age groups, Table 6 presents the ADI values calculated according to body mass of the respondent.

**Figure 5:**
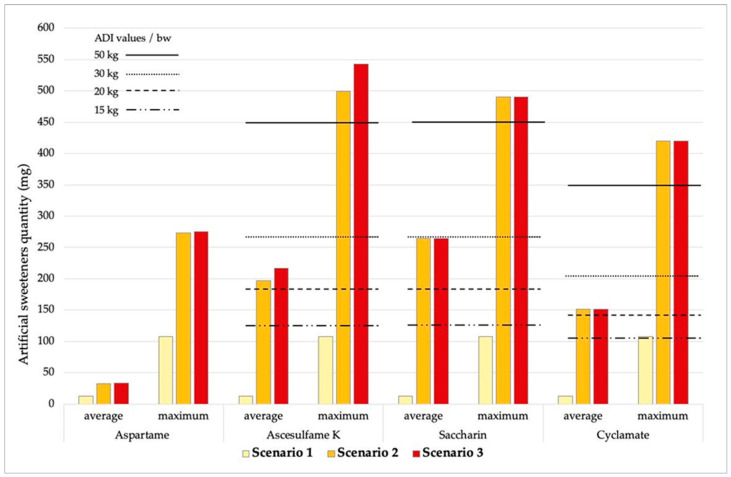
Presentation of different consumption scenarios based on average and highest artificial sweeteners values in the analyzed products, compared to the acceptable daily intake for subjects of different ages and body weights.

A summary of results from the different consumption scenarios (Table 3, Table 4 and Table 5) is shown in Figure 5. The figure compares the estimated intake of ASs (both average and maximum values) in the analyzed products with ADI values, adjusted for the body weight of the participants. The ADI values for aspartame across all three consumption scenarios are not visible in the figure because the ADI threshold is relatively high at 40 mg kg^−1^.

### 3.3. Assessment of Product Labeling

The labels of all samples were reviewed in order to verify whether they meet the requirements of Regulation 1169/2011 on consumer information about food. The results are shown in Table 7.

## 4. Discussion

The results of analyses have clearly indicated a high prevalence of ASs across various food products, frequently consumed by respondents. Notably, some of these products— particularly soft drinks—have been identified as major contributors to excessive sugar intake in these age groups, which is associated with the occurrence of overweight and obesity [43,44]. In response, public health initiatives have been implemented to reduce sugar consumption, including educational campaigns, warnings about adverse effects of sugar, and the introduction of special taxes on the sugar content in soft drinks. As a result of these measures, ASs are now incorporated into a broader range of food products. This increasing presence raises important questions regarding dietary intake, particularly the potential cumulative effects of consuming multiple ASs simultaneously, as many products contain two or more ASs in combination. Calculated exposure assessment relies on available data regarding the concentration of these substances in different food categories, alongside dietary consumption data collected through questionnaires.

### 4.1. Food Product Labeling

According to legal regulations, information on the presence of sweeteners must be displayed prominently alongside the product name, ensuring that consumers receive complete and easily accessible information about the product’s composition [17]. The product name is typically displayed on the front of the package, within the principal field of vision, which the consumer first notices. According to the Regulation 1169/2011, “principal field of vision means the field of vision of a package which is most likely to be seen at first glance by the consumer at the time of purchase and that enables the consumer to immediately identify a product in terms of its character or nature”. Furthermore, Annex III of the same regulation mandates that the statement “with sweetener(s)” must accompany the product name [17].

An analysis of the sampled products revealed inconsistencies in labeling practices. Among the 60 samples in which ASs were detected, only five were visibly marked with the “zero” label—a commonly recognized indicator for sugar-free products containing ASs. The remaining products failed to include a clear statement about added sweeteners along with the name highlighted in the principal field of the label, as required by regulations. A detailed examination of product declarations revealed that in 54 samples containing ASs, their presence was only disclosed in the list of ingredients on the back of the packaging, rather than being prominently displayed alongside the product name. Notably, in one fruit nectar sample, the artificial sweetener cyclamate was entirely absent from the ingredient list, raising concerns about labeling accuracy and transparency.

Regarding the labeling of high-protein milk products containing ASs, none included this information near product name in the principal field of vision. However, the packaging prominently highlighted attributes such as reduced fat content, protein quantity, and lactose-free claims.

For most consumers, the principal field of vision and information displayed there play a crucial role in product selection and purchasing decisions [45,46,47]. Visual cues, such as imagery and branding, also significantly influence consumer perception. Notably, fruit images are commonly featured on both fruit juices and nectars, which may contribute to consumer confusion regarding product differences. A 2022 survey conducted in the Republic of Croatia, including 415 respondents, revealed that most participants were unaware of the distinction between fruit juice and nectar [48]. Similar findings have been reported in studies from other countries [49,50,51], highlighting a widespread lack of awareness that could further affect consumer understanding of product composition, including the presence of ASs.

Our survey results further highlight the gap between labeling regulations and consumer awareness. Most of the respondents—51.2% of parents and 70.8% of university and secondary school students—reported that they rarely or never read product labels [32]. These findings align with the previous studies on consumer attitudes towards label reading [16,52,53,54]. However, despite their limited engagement with labeling information, a substantial percentage of respondents—74% of parents and 54.2% of university and secondary school students—perceived ASs as potentially harmful and have an adverse attitude towards them. At the same time, the survey revealed high consumption rates of beverages and dairy products containing ASs. Specifically, 30.2% of parents stated that their children often drink store-bought fruit drinks, compared to 49.2% of university and secondary school students [31]. Similarly, the consumption of high-protein milk drinks was notable, with 13.3% of parents reporting that their children often consume them (7.7% every day), while 39.7% of university and secondary school students often consumed these products [32].

These findings highlight a clear discrepancy between consumer concerns about ASs and their actual dietary intake, suggesting that unintentional consumption may be driven by inadequate labeling practices and limited awareness. Given the results of the sample analyses, respondents’ perceptions of ASs, and their reported habits regarding label reading, it is reasonable to conclude that many consumers remain unaware that the food products they consume daily contain ASs.

This highlights the urgent need for stricter enforcement of labeling regulations and improved consumer education on food label literacy. Ensuring that ASs are clearly and consistently disclosed on packaging could help consumers make more informed dietary choices, particularly those who actively seek to avoid or limit their intake of such additives. To address this issue, targeted public health initiatives should be implemented to enhance consumer awareness and promote food label literacy. Educational campaigns should prioritize delivering clear, evidence-based information about ASs, equipping consumers with the knowledge necessary to navigate product labeling effectively and make choices aligned with their health preferences.

### 4.2. Artificial Sweetener Intake

ASs were detected in a large number of food products analyzed in our study, specifically in 67.8% of the samples. The majority of these products contained a combination of two or more ASs, with acesulfame-K and sucralose being the most commonly used combination. This widespread practice enhances the perceived sweetness while masking undesirable taste characteristics, such as bitterness or sourness, associated with certain sweeteners [55,56,57]. Additionally, the use of multiple sweeteners ensures compliance with regulatory limits set by Regulation 1333/2008 on food additives, as it prevents exceeding the maximum allowable concentrations of any single sweetener. Notably, in some of the analyzed samples, the concentration of individual AS was very slightly below the prescribed limit. However, these products also contained other sweeteners, highlighting the cumulative presence of multiple additives within a single product.

High-protein milk products have gained popularity among children and young people, often being marketed as healthy options due to their high-protein content. However, studies suggest a potential link between higher animal protein intake during childhood and an increased BMI, as excess protein also contributes additional calories [58,59]. Moreover, these products frequently contain significant amounts of sugar, further elevating their energy value. For that reason, very often in these products sugar is replaced with ASs.

Our analysis of 20 high-protein milk products—including drinks, puddings, and skyr products—revealed that 14 samples contained one or more ASs. The combination of two sweeteners, acesulfame-K and sucralose, was the most common prevalent (in 9 out of 14 samples). Notably, one high-protein pudding sample contained three ASs, two of which (acesulfame-K and cyclamate) exceeded the maximum allowed concentrations. Also, the majority of analyzed beverages (67%) contained multiple ASs, highlighting the prevalent industry practice of using sweetener combinations to enhance taste while reducing sugar content.

Opinions about the advantages or disadvantages of ASs consumption remain a subject of ongoing debate. While a considerable number of studies indicate that the ASs can have positive effects, such as preventing caries [60,61], aiding in weight regulation [62], and reducing the risk of cardiovascular and metabolic diseases [24,63], there are also studies that challenge these claims and disprove the benefits. Critics argue that the potential risks of ASs may outweigh their benefits, highlighting concerns that include possible negative impacts on health [24,25,29,30,64,65,66,67].

Due to their lower body weight and relatively high fluid and food intake compared to adults, children consume larger quantities of substances per kg of body weight [68]. The widespread use of ASs in various food products complicates the assessment of their dietary intake and comparison with the acceptable daily intake values. Hypothetical consumption scenarios (Figure 5) clearly demonstrate that children, with the lowest body mass, are particularly susceptible to the potential adverse effects of high ASs intake. Given that the ADI is expressed per kilogram of body weight, and considering the type of ASs consumed, it is not uncommon for children to approach or exceed the recommended ADI thresholds. Our study included 95 children aged 2–6 years, with a body weights between 15 and 19.5 kg, and 118 children aged 4–8 years, with a body weight between 20 and 25 kg. These age and weight groups represent a particularly vulnerable population, emphasizing the need for careful consideration of ASs consumption during early childhood.

The results obtained for aspartame across all three consumption scenarios indicate that reaching the ADI threshold is unlikely, given that the ADI for aspartame is relatively high at 40 mg kg^−1^. In our study, which included 32 children aged 5–8 years with body weights ranging from 23.5 to 25 kg, the calculated ADI for aspartame ranged from 940 to 1000 mg. Considering the concentrations of aspartame found in various food product included in the proposed consumption scenarios, it is evident that aspartame itself does not pose a significant risk to this population.

When considering acesulfame-K, saccharin, or cyclamate, the data raise great concerns. Unlike aspartame, these ASs have lower ADI values, making it more likely for children with lower body weight to exceed the ADI threshold.

Specifically, children weighing 15 and 20 kg who consume products in quantities corresponding to Scenario 2 or 3 will surpass the ADI threshold, regardless of whether they consume products containing the average or highest concentrations of these ASs. Furthermore, if products with the highest measured concentrations are consumed in the amounts outlined in Scenario 2 or 3, even children with a body weight of 30 kg, as well as individuals weighing 50 kg, will exceed the ADI limit. Notably, in the case of cyclamate, children with a body weight of 15 kg may exceed the ADI threshold even when consuming products with the highest detected concentrations in smaller quantities, as represented in Scenario 1. These findings highlight the potential risks of exceeding the ADI values due to the excessive consumption of artificial-sweetener-containing food products, particularly among children with lower body weight. Some studies apply higher ADI values for acesulfame-K, saccharin, and cyclamate, compared to the EFSA values which were used in this study [25]. However, although higher ADI values may result in slower exceedances, the risk to low-weight children remains considerable.

It is important to note that the potential health risks associated with these artificial sweeteners are still a topic of debate, and while some studies have raised concerns, most regulatory bodies maintain that these sweeteners are safe for human consumption at levels within the established ADI. However, as our study suggests, the cumulative intake of multiple ASs, often combined in various food products, may warrant closer scrutiny, particularly in vulnerable populations such as children. Therefore, given the increasing prevalence of ASs in various food categories, understanding their current exposure is essential to inform public health recommendations and future research directions.

While this study primarily quantifies the concentrations of ASs in commonly consumed food products, understanding their potential health impacts requires a broader perspective that includes their metabolic effects. Although our research does not directly investigate these effects, our findings provide valuable insight into population-level exposure to ASs. The widespread consumption of these sweeteners, particularly when present across multiple food categories, raises concerns about potential metabolic implications, highlighting the need for further studies to assess these impacts more comprehensively.

While this study also did not focus on long-term effects, we recognize the importance of this research gap and highlight the need for further studies that investigate the long-term health effects of ASs consumption. Future studies should focus on how excessive intake of ASs affects metabolic pathways and whether such changes contribute to long-term health risks such as obesity, metabolic disorders, and other chronic diseases over extended periods. Gaining a deeper understanding of these mechanisms is essential to fully understand the scope of ASs exposure and its possible role in shaping health outcomes.

Also, it is essential to drive changes in both food production and consumer habits. One approach is to encourage manufacturers to increase the fruit content in juice-based beverages, which can enhance the sensory profile while reducing the need for added sugars or artificial sweeteners. A study conducted in Poland demonstrated the effectiveness of such an approach, as the quality of fruit juice-based beverages improved significantly following the introduction of a tax on added sugar. This policy led to a reduction in added sugars and an increase in fruit content, highlighting the potential of regulatory measures to drive positive change [69]. Another alternative is the increased use of natural sweeteners, which may offer a more acceptable substitute while maintaining sweetness perception [70].

### 4.3. Study Strenghts and Limitations

This study has several limitations that should be considered when interpreting the results. Additionally, this study focused on a limited selection of commonly consumed food products and beverages from Croatian markets. Future research should consider expanding both the sample size and the range of food categories to better capture the diversity of AS in the diet.

Moreover, various confounding factors—such as age, gender, environment, lifestyle, and economic status—may influence taste preferences, dietary habits, and food choices, which could, in turn, affect consumption patterns and intake of ASs. Although we did not analyze these variables in the current study, in a parallel study, we explored consumer perceptions of artificial sweeteners, their consumption frequency, and potential effects on BMI, considering various socio-demographic factors [32]. We acknowledge their potential impact and recommend that future research account for these factors to provide a more comprehensive understanding of how demographic and socio-economic factors impact estimated ASs intake levels.

Despite these limitations, our findings, although primarily specific to the Republic of Croatia, offer valuable insights that can serve as a reference for similar studies in countries with comparable dietary habits and food regulations. While the sample may not fully represent the broader Croatian population, it offers valuable data for understanding ASs exposure, particularly within specific subgroups and consumption patterns. Expanding research to a wider range of countries would contribute to a more comprehensive global perspective on ASs consumption.

One of its key strengths is the detailed quantification of AS in commonly consumed food products, providing valuable insight into population-level exposure. By analyzing multiple ASs across various food categories, the study offers a more comprehensive perspective on dietary intake patterns, which is crucial for assessing potential health risks.

Additionally, this study utilizes a well-defined methodology that ensures the accuracy and reliability of the data. The inclusion of a sample population that reflects real-world consumption habits strengthens the relevance of the findings.

Another significant strength is this study’s contribution to identifying potential areas of concern, particularly for vulnerable populations such as children. By highlighting the cumulative intake of multiple ASs, this research underscores the need for further investigation into long-term metabolic and health effects. These findings can help inform public health policies and future dietary guidelines aimed at ensuring safe ASs consumption.

## 5. Conclusions

To our knowledge, this is the first study in Croatia to quantify the content of ASs in various frequently consumed food products and assess the dietary intake for the targeted groups. A key finding of this study is the simultaneous presence of multiple AS within the same product, highlighting a gap in current research regarding their cumulative intake. While existing evidence predominately focuses on individual effects of different types of ASs, there is a lack of studies addressing their cumulative intake. Consequently, the conventional approach of accessing ASs intake based on individual ADI values may not fully capture the complexities of cumulative exposure.

Given these findings, it is essential to adopt a broader perspective on ASs consumption and encourage further research into their long-term metabolic and health effects. Collaboration among health professionals, policymakers, and educators is crucial in raising awareness of the potential risks of exceeding the ADI values and promoting informed dietary choices, particularly among children and other vulnerable populations.

## Figures and Tables

**Table 1:** Distribution and concentrations of artificial sweeteners across various food product categories analyzed in this study. Values are expressed in mg L^−1^, except for chewing gums, which are expressed in mg kg^−1^.

		Aspartame (mg L^−1^)		Acesulfame-K (mg L^−1^)		Saccharin (mg L^−1^)		Cyclamate (mg L^−1^)	
Food Product	n	$\bar{x}$	Range	$\bar{x}$	Range	$\bar{x}$	Range	$\bar{x}$	Range
Energy drinks	7	0.0	<LOQ	80.0	<LOQ–207.1	0.0	<LOQ	0.0	<LOQ
Carbonated and non-carbonated soft drinks	47	26.6	<LOQ–493.6	33.8	<LOQ–268.7	5.4	<LOQ–63.1	33.6	<LOQ–241.0
Instant drinks	6	0.0	<LOQ	52.0	<LOQ–312.0	103.8	<LOQ–622.7	28.8	<LOQ–172.7
Juices/nectars	26	<LOQ	<LOQ–10.2	4.7	<LOQ–74.4	4.2	<LOQ–33.9	15.8	<LOQ–129.3
High-protein drinks	20	0.0	<LOQ	70.9	<LOQ–352.0 **	0.0	<LOQ	13.2	<LOQ–264.6 **
Syrups	3	24.2	10.4–37.1	121.0	10.0–129.7	201.0	150.5–250.4	131.7	125.6–138.2
Chewing gums	12	573.0	<LOQ–1791.5	652.7	<LOQ–1520.4	0.0	<LOQ	0.0	<LOQ

$\bar{x}$—average value; LOQ—limit of quantitation = 1 mg L^−1^; ** results higher than the maximum permitted level in Regulation 1333/2011 on food additives.

**Table 2:** Concentrations of artificial sweeteners, average and maximum values, including only samples in which artificial sweeteners were found. Values are expressed in mg L^−1^, except for chewing gums, which are expressed in mg kg^−1^.

		Aspartame (mg L^−1^)		Acesulfame-K (mg L^−1^)		Saccharin (mg L^−1^)		Cyclamate (mg L^−1^)	
Food Product	n	$\bar{x}$	Max	$\bar{x}$	Max	$\bar{x}$	Max	$\bar{x}$	Max
Energy drinks	6	0.0	0.0	93.3	207.1	0.0	0.0	0.0	0.0
Carbonated and non-carbonated soft drinks	35	35.7	493.6	45.3	268.7	7.3	63.1	44.1	241.0
Instant drinks	2	0.0	0.0	156.0	312.0	311.4	622.7	86.4	172.7
Juices/nectars	14	1.9	10.2	8.5	74.4	7.8	33.9	29.3	129.3
High-protein drinks	14	0.0	0.0	101.2	350.2	1.3	17.9	18.9	264.6
Syrups	3	24.2	37.1	121.0	129.7	201.0	250.4	131.7	138.2
Chewing gums	8	858.8	1791.5	976.3	1520.4	0.0	0.0	0.0	0.0

$\bar{x}$—average value; Max—maximum value.

**Table 3:** Estimated daily intake of artificial sweeteners based on Scenario 1.

		Aspartame (mg)		Acesulfame-K (mg)		Saccharin (mg)		Cyclamate (mg)	
Food Product	Volume (L)/Quantity	$\bar{x}$	Max	$\bar{x}$	Max	$\bar{x}$	Max	$\bar{x}$	Max
Energy drinks	0	0.00	0.00	0.00	0.00	0.00	0.00	0.00	0.00
Carbonated and non-carbonated soft drinks	0.2	7.14	98.72	9.06	53.74	1.46	12.62	8.82	48.20
Instant drinks	0.2	0.00	0.00	31.20	62.40	62.28	124.54	17.28	34.54
Juices/nectars	0.2	0.38	2.04	1.70	14.88	1.56	6.78	5.86	25.86
High-protein drinks	0.15	0.00	0.00	15.18	52.53	0.20	2.69	2.84	39.69
Syrups	0.2	4.84	7.42	24.20	25.94	40.20	50.08	26.34	27.64
Chewing gums/pcs.	0	0.00	0.00	0.00	0.00	0.00	0.00	0.00	0.00
Estimated daily intake (mg)		12.36	108.18	81.34	209.49	105.70	196.71	61.14	175.93

$\bar{x}$—average value; Max—maximum value; pcs.: refers to the individual piece of chewing gum.

**Table 4:** Estimated daily intake of artificial sweeteners based on Scenario 2.

		Aspartame (mg)		Acesulfame-K (mg)		Saccharin (mg)		Cyclamate (mg)	
**Food Product**	Volume (L)/Quantity	$\bar{x}$	Max	$\bar{x}$	Max	$\bar{x}$	Max	$\bar{x}$	Max
Energy drinks	0	0.00	0.00	0.00	0.00	0.00	0.00	0.00	0.00
Carbonated and non-carbonated soft drinks	0.5	17.85	246.80	22.65	134.35	3.65	31.55	22.05	120.50
Instant drinks	0.5	0.00	0.00	78.00	156.00	155.70	311.35	43.20	86.35
Juices/nectars	0.5	0.95	5.10	4.25	37.20	3.90	16.95	14.65	64.65
High-protein drinks	0.3	0.00	0.00	30.36	105.06	0.39	5.37	5.67	79.38
Syrups	0.5	12.10	18.55	60.50	64.85	100.50	125.20	65.85	69.10
Chewing gums/pcs.	1	1.20	2.51	1.37	2.13	0.00	0.00	0.00	0.00
Estimated daily intake (mg)		32.10	272.96	197.13	499.59	264.14	490.42	151.42	419.98

$\bar{x}$—average value; Max—maximum value; pcs.: refers to the individual piece of chewing gum.

**Table 5:** Estimated daily intake of artificial sweeteners based on Scenario 3.

		Aspartame (mg)		Acesulfame-K (mg)		Saccharin (mg)		Cyclamate (mg)	
Food Product	Volume (L)/Quantity	$\bar{x}$	Max	$\bar{x}$	Max	$\bar{x}$	Max	$\bar{x}$	Max
Energy drinks	0.2	0.00	0.00	18.66	41.42	0.00	0.00	0.00	0.00
Carbonated and non-carbonated soft drinks	0.5	17.85	246.80	22.65	134.35	3.65	31.55	22.05	120.50
Instant drinks	0.5	0.00	0.00	78.00	156.00	155.70	311.35	43.20	86.35
Juices/nectars	0.5	0.95	5.10	4.25	37.20	3.90	16.95	14.65	64.65
High-protein drinks	0.3	0.00	0.00	30.36	105.06	0.39	5.37	5.67	79.38
Syrups	0.5	12.10	18.55	60.50	64.85	100.50	125.20	65.85	69.10
Chewing gums/pcs.	2	2.40	5.02	2.73	4.26	0.00	0.00	0.00	0.00
Estimated daily intake (mg)		33.30	275.47	217.15	543.14	264.14	490.42	151.42	419.98

$\bar{x}$—average value; Max—maximum value; pcs.: refers to the individual piece of chewing gum.

**Table 6:** Acceptable daily intake values calculated according to body mass of the respondent.

	Aspartame ADI (40 mg kg^−1^)	Ascesulfame-K ADI (9 mg kg^−1^)	Saccharine ADI (9 mg kg^−1^)	Cyclamate ADI (7 mg kg^−1^)
	ADI Values According to Body Mass			
Child with a body mass of 15 kg	600	135	135	105
Child with a body mass of 20 kg	800	180	180	140
Child with a body mass of 30 kg	1200	270	270	210
Person with a body mass of 50 kg	2000	450	450	350
Person with a body mass of 70 kg	2800	630	630	490

ADI—acceptable daily intake.

**Table 7:** Assessment of labels compliance with Regulation 1169/2011 on food information to consumer (part related to sweeteners).

Food Product	n	Samples with ASs (n)	Samples with AS Stated in the Principal Field of the Label (n)	Incorrect Labeling of ASs in Food Products (%)
Energy drinks	7	6	4	33.3
Carbonated and non-carbonated soft drinks	47	35	5	85.7
Instant drinks	6	2	1	50.0
Nectars/non-carbonated fruit drinks	23	14	1	92.9
Juices	3	0	/	/
High-protein milk products	20	14	0	100.0
Syrups	3	3	0	100.0
Chewing gums	12	8	8	0.0

ASs—artificial sweeteners.

## Data Availability

The raw data supporting the conclusions of this article will be made available by the authors on request due to legal constrictions.

## Abbreviations

ADI	acceptable daily intake
AS	artificial sweetener
BMI	Body Mass Index
EFSA	European Food Safety Authority
HPLC	High Pressure Liquid Chromatography
LOQ	limit of quantitation
QS	quantum satis (QS)
LSC	Low-Caloric Sweetener
NSC	Non-Caloric Sweetener
